# Diagnostic value and immune microenvironment regulatory network of metabolic reprogramming in chronic rhinosinusitis with nasal polyps identified by multidimensional transcriptome integration and machine learning

**DOI:** 10.3389/fimmu.2026.1808799

**Published:** 2026-05-25

**Authors:** Li Zhao, Xiang Jiang Meng, Xu Liang, Guang Mei Yuan, Li Shi

**Affiliations:** 1 Department of Otolaryngology, The Second Qilu Hospital of Shandong University, Jinan, China; 2 Department of Medical Equipment, Yishui County People’s Hospital, Yishui, China

**Keywords:** bioinformatics analysis, chronic rhinosinusitis with nasal polyps, machine learning, Mendelian randomization, metabolic reprogramming, single-cell

## Abstract

**Background:**

Chronic Rhinosinusitis with Nasal polyps (CRSwNP) are characterized by chronic inflammation and occur in 1–4% of the population worldwide. Patients often have comorbid asthma, and standard treatments among them are hindered by significant recurrence and lack of durability. Currently, knowledge of the molecular circuitry and immune microenvironmental interplay that utilizes metabolic reprogramming within CRSwNP is incomplete.

**Methods:**

Utilizing CRSwNP datasets from the GEO database, we performed bioinformatics analysis to identify differentially expressed genes (DEGs) implicated in metabolic reprogramming. Key regulatory genes were subsequently selected by weighted gene co-expression network analysis (WGCNA) and machine learning algorithms; their relationship with the immune microenvironment was then evaluated. To further investigate the underlying pathogenic mechanisms, we performed single-cell RNA sequencing (scRNA-seq) to map cellular expression patterns and applied Mendelian randomization (MR) analysis to assess potential causal relationships. Key molecules were subsequently experimentally validated by quantitative real-time PCR (qRT-PCR).

**Results:**

We identified 21 DEGs associated with metabolic reprogramming that are relevant to CRSwNP. This subset was then analyzed using machine learning to identify 8 hub genes - ERBB4, FBP1, HMGCS2, LYZ, NDRG2, PIP, PYCR1, and SLC43A1. A prediction model built using these biomarkers yielded high diagnostic performance (AUC = 0.979). Single-cell resolution analysis revealed that distinct expression patterns were exhibited by these genes across subsets of immune cells. MR analysis determined that lower expression of FBP1, LYZ and NDRG2 could be risk factors for CRSwNP. Subsequent qRT-PCR in independent samples validated the downregulation of these genes in CRSwNP tissues.

**Conclusions:**

We systematically identify and validate a set of metabolic reprogramming-related genes with diagnostic value in CRSwNP. Collectively, these findings not only heighten the current mechanistic understanding of CRSwNP pathogenesis but also offer a novel platform to devise diagnostic and therapeutic avenues focusing on metabolism.

## 1 Background

Chronic Rhinosinusitis with Nasal polyps (CRSwNP) is a common chronic inflammatory disease of the nose manifested by persistent nasal obstruction, hyposmia and increased secretions, which in extreme cases leads to sinonasal dysfunction and associated complications, significantly reducing patients’ quality of life (1). Many factors probably contribute to the increasing incidence of CRSwNP. In addition, the disease is frequently recurrent and refractory, leading to a significant burden on individuals and the health care system (2). With a worldwide prevalence of about 1%-4%, CRSwNP are often associated with asthma and allergic rhinitis complicating their clinical management (3).

Current standard of care is still largely dominated by corticosteroids and surgical approach, however, both have been hampered by high rates of recurrence and poor long-term outcomes in many cases (1). While biologic therapies may provide a more recent option for some, usage is limited by expense and varied treatment responses (4). Current diagnostics rely primarily on imaging, endoscopic and symptom scoring. However, a major gap exists in that there are no molecular tools for early diagnosis or subtyping of disease that would ultimately help drive precision medicine forward (5). Given these limitations, there is a growing need to determine the deeper molecular mechanisms driving CRSwNP, revealing an obvious clinical applicability in terms of novel biomarker discovery.

Recent studies indicate the mechanisms by which metabolic reprogramming enables cells to adapt to changes to the microenvironment. This process generally drives the progression of chronic inflammatory disease and tumors, primarily through the regulation of energy supply, transduction and the immune response (6). Accordingly, scRNA-seq has revealed that a striking feature of CRSwNP is the upregulation of glycolytic enzyme genes within particular cell types—most notably basal epithelial cells, stromal cells and memory T cells (7). This suggests a marked activation of aerobic glycolysis (the Warburg effect) in this condition. HIF-1α, an important glycolytic regulator, is upregulated in CRS. Increased expression of HIF-1α appears to promote the differentiation of fibroblasts to myofibroblasts and promotes extracellular matrix production, suggesting that glycolytic reprogramming likely drives tissue remodeling in CRSwNP (8).

Lipid metabolic disorder is another key feature of CRSwNP, wherein the specific accumulation of long-chain fatty acids, especially unsaturated ones like arachidonic acid, occurs in the polyp. The accumulation of these lipids, specifically long-chain unsaturated fatty acids, in the airway inflammatory microenvironment drives eosinophil extracellular trap formation and corticosteroid resistance through the IRE1α/XBP1s/PAD4 axis contributing to the refractory nature of the disease (9). Metabolomics also showed enhanced oxidation of long-chain unsaturated fatty acids in polyp tissue positively correlating with eosinophil infiltration and IL-5 mRNA (10).

However, studies have not provided a comprehensive description of the molecular networks and regulatory mechanisms of metabolic reprogramming in CRSwNP, and their interactions with the immune microenvironment, precluding a comprehensive understanding of disease mechanisms and the development of metabolism-targeted diagnostic and therapeutic strategies.

Therefore, this study aims to systematically analyze the molecular characteristics related to metabolic reprogramming in CRSwNP. We first analyzed public transcriptomic and scRNA-seq data and integrated multi-dimensional bioinformatics approaches—including WGCNA, differential expression analysis, functional enrichment analysis, and immune infiltration assessment—to systematically screen key hub genes associated with metabolic reprogramming and the immune microenvironment. Feature selection was performed using machine learning, followed by cross-validation across multiple datasets and independent cohort validation to construct a molecular signature and risk model with diagnostic value. This study is expected to provide new theoretical foundations and potential tools for molecular subtyping, early precise diagnosis, and targeted therapy development of CRSwNP from a metabolic perspective. These advances could promote mechanistic research and facilitate clinical translation in this field.

## 2 Methods

### 2.1 Data sources and preprocessing

**Figure 1:**
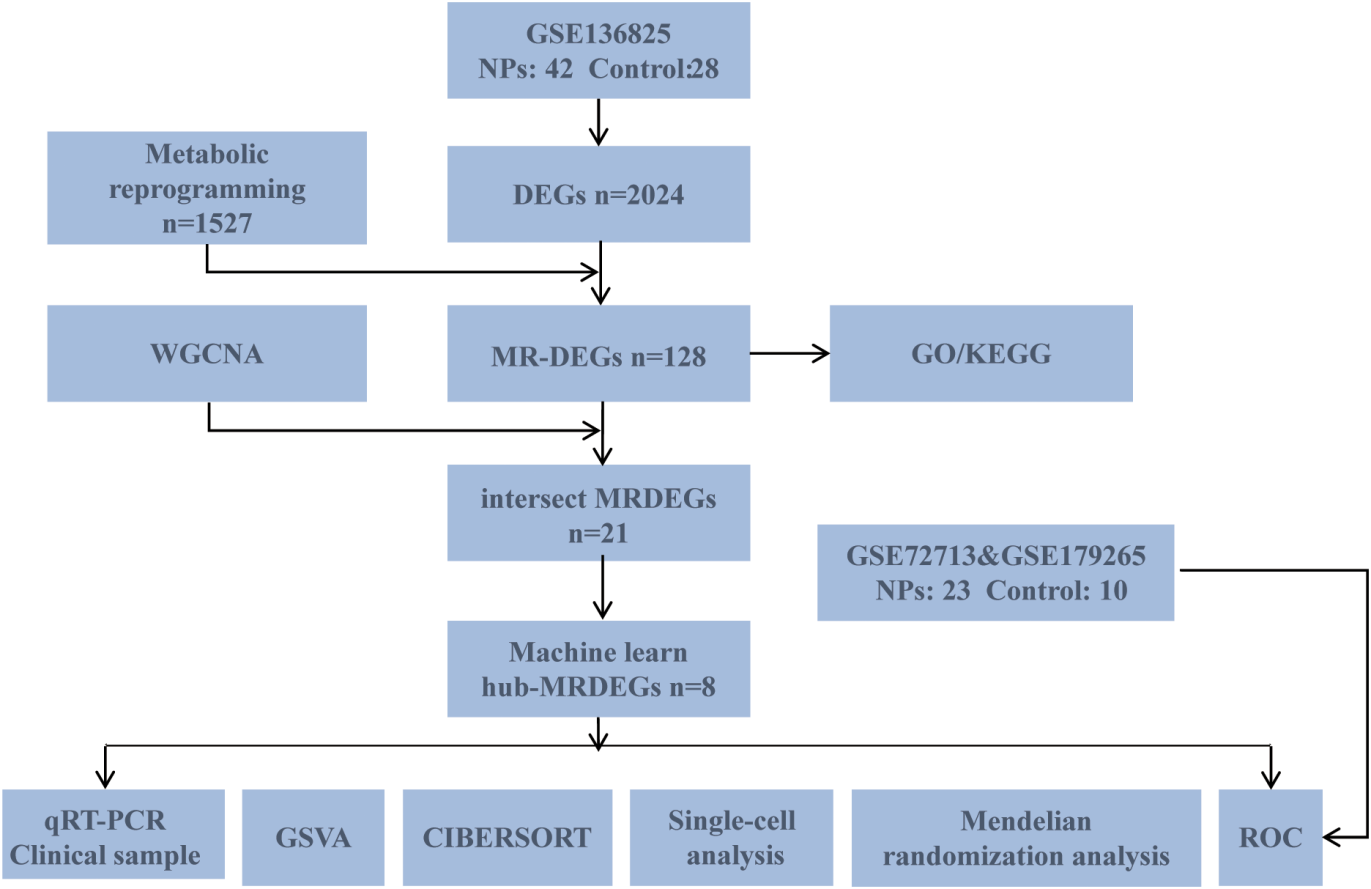
Flowchart of the study design.

We acquired transcriptomic data for CRSwNP and control tissues systematically from the Gene Expression Omnibus (GEO) database. All data were publicly available and anonymized, requiring no new ethical approval. The specific datasets are as follows: Training Set: GSE136825 (based on platform GPL20301), containing 42 CRSwNP tissues and 28 normal nasal mucosa control tissues. Validation Set: GSE72713 and GSE179265 were merged, resulting in a combined set of 23 CRSwNP tissues and 10 normal control tissues for external validation of the model. scRNA-seq Data: GSE202100 (based on platform GPL24676, Illumina NovaSeq 6000 sequencing), containing 5 CRSwNP samples for dissecting cellular heterogeneity. The overall study design is summarized in **Figure 1**.

### 2.2 Screening of differentially expressed genes and metabolic reprogramming related genes

To identify differentially expressed genes (DEGs) in CRSwNP, we performed differential expression analysis on the counts data of dataset GSE136825 using the DESeq2 R package (version 1.42.1). We defined significant DEGs using a threshold of FDR-adjusted *P* < 0.05 and |log2FC| > 1. These DEGs were subsequently visualized in volcano plots generated using the R package ggplot2.

To obtain a set of relevant genes, we extracted metabolic reprogramming related genes from GeneCards database (Version 5.24) using the search term “Metabolic Reprogramming” and filtering for genes with a relevance score >10, generating a preliminary set of 1527 genes (11). This threshold effectively enriches substantially functionally associated genes while excluding indirect or weakly associated entries, balancing core gene capture with background noise control. The union between these metabolic reprogramming related genes and genes from our DEGs was taken, obtaining our Metabolic Reprogramming-related differentially expressed genes (MR-DEGs), as shown in the Venn diagram.

### 2.3 Functional and pathway enrichment analysis

Functional enrichment analysis was then performed to uncover the biological roles and pathway associations of the MR-DEGs without stratifying genes by up- or down-regulation. We applied both Gene Ontology (GO) and Kyoto Encyclopedia of Genes and Genomes (KEGG) for the interpretation of the gene list (12). GO annotation encompasses three main types of information: The Biological Process (BP) domain addresses the larger biological programs involving one or more molecular activities; the Cellular Component (CC) domain describes the specific locations within cells where gene products are active; and the Molecular Function (MF) domain describes the specific biochemical activities of individual gene products. This approach captures interactions at multiple levels of the biological programming. Terms below the threshold of FDR < 0.05 were selected as having significant enrichment, and the top 10 enriched terms from each category are plotted as bar plots.

### 2.4 Construction of protein-protein interaction network

To evaluate whether any of the MR-DEGs interact with each other, we assembled a protein-protein interaction (PPI) network using STRING database (https://cn.string-db.org), focusing on the overlapping co-expressed MR-DEGs. The resulting network was imported into and visualized with Cytoscape software. Only interactions with a confidence score≥0.7 were retained.

### 2.5 Weighted gene co-expression network analysis

We performed WGCNA (12) to determine systematic co-expression of genes and their relationship to CRSwNP. This was implemented using WGCNA R package (version 1.73) (13). This first step involved subsetting the top 20% of genes (4947 genes) having the highest median absolute expression across all samples in the discovery set. After removing outlier samples by clustering, we used the pickSoftThreshold function to select soft-thresholding power (β) of 4 for a scale-free topology fit index (R^2^) of 0.85. This yielded an adjacency matrix and a Topological Overlap Matrix (TOM). Modules of correlated genes were identified using the dynamic tree cut method, with a minimum module size set to 50 genes and a merge cut height threshold of 0.25.

To identify those modules most strongly associated with the disease, we calculated the correlation between all module eigengenes (ME) and the CRSwNP phenotype, selecting for subsequent analysis the key module with strongest association to the disease.

### 2.6 Cross-screening of key genes and refinement via machine learning

Core candidate genes were defined through the intersection of genes from the key WGCNA module and MR-DEGs, yielding a total of 21 genes. To refine the above gene set into a smaller and more robust panel of biomarkers for CRSwNP, we employed six machine learning algorithms for feature selection. The purpose of this analysis was to achieve biomarker screening based on multi−algorithm consensus, rather than a direct competition of model performance.

Bagging with Decision Trees: For more complex learners such as decision trees, we can apply bagging. Here the learner is now a decision tree that is trained on numerous bootstrap samples of the data and as always the individual predictions are then aggregated (14). Bayesian algorithms combine prior beliefs and knowledge with the empirical data collected by experiment using Bayes’ theorem. Bayesian algorithms can update posterior probability estimates and are useful in estimates of model parameters, in choosing the most likely model, and in making predictions (15).LVQ Algorithm: Through competitive learning, prototype vectors representing different classes are generated in the feature space, and patterns are classified based on the nearest neighbor principle (16). However, this algorithm is suitable for small-scale datasets and has limited scalability for large-scale data. Least Absolute Shrinkage and Selection Operator (LASSO) Regression: The glmnet package selected genes with non-zero coefficients via 10-fold cross-validation using the lambda value that minimized model deviance (17). Random Forest (RF): Using the randomForest package, gene importance was assessed based on the Gini impurity decrease. Genes with importance scores in the top 20% were selected (18). Boruta Algorithm: Using the Boruta package, genes with importance significantly higher than that of random shadow features were selected.

To minimize algorithm−specific false positives, only genes identified by at least five of the six algorithms were retained. This consensus approach captures core molecules that are robust across multiple methods, balancing sensitivity and specificity to enhance biomarker reproducibility. These genes were defined as hub metabolic reprogramming differentially expressed genes (hub−MRDEGs) for subsequent analyses.

### 2.7 Diagnostic model construction and validation

Wilcoxon rank-sum tests were performed to compare the eight hub−MRDEGs between the CRSwNP and control groups, and violin plots were generated to visualize the differentially expressed feature genes. To evaluate the predictive model, a nomogram for CRSwNP risk was constructed using the rms package based on the expression signatures of hub−MRDEGs, where the score weight for each gene was derived from the regression model coefficients. All genes were entered as continuous variables, and the total score of the nomogram corresponds to the probability of developing CRSwNP (19). The calibration curve generated by 1000 bootstrap resamples assessed the agreement between model predictions and actual outcomes. Subsequently, decision curve analysis (DCA) was performed to quantify clinical net benefit, while the discriminatory power was validated by generating receiver operating characteristic curves and computing the AUC value using the pROC package (20). Finally, the model’s discrimination and calibration were externally validated using the independent validation set (merged GSE72713 and GSE179265).

### 2.8 Immune infiltration analysis and correlation study

We employed the CIBERSORT algorithm with the LM22 signature matrix to deconvolute bulk RNA-seq data and estimate the relative proportions of 22 immune cell subtypes in each sample (21). The Wilcoxon rank-sum test was employed to assess differences in immune cell composition between the CRSwNP and control groups. Spearman correlation analysis was performed to explore associations between hub gene expression levels and the infiltration levels of specific immune cells.

### 2.9 Single-cell transcriptomic data analysis

The scRNA-seq data were processed using the Seurat package (version 5.3.0) (22). Quality control criteria were: number of genes detected per cell between 200 and 5000, and mitochondrial gene percentage below 20%. Potential doublets were identified and removed using the DoubletFinder package. The data were logarithmically normalized, and the 2000 most highly variable genes were selected for principal component analysis (PCA). The Harmony package was used for batch effect correction. A K-nearest neighbor graph was constructed based on the top 18 principal components, followed by clustering (resolution = 0.8) and visualization using Uniform Manifold Approximation and Projection (UMAP). Cell types were annotated using the SingleR package with the human primary cell atlas as a reference, followed by manual verification based on known marker genes. Subsequently, we performed differential expression analysis via the FindMarkers function, using a threshold of |log2FC| > 0.5 and an adjusted *P*-value < 0.05.

### 2.10 MR analysis of gene expression and CRSwNP

Two-sample MR was performed to elucidate whether alterations in gene expression exhibit a causal relationship with the development of CRSwNP (23). Instrumental variables (IVs) for exposure data (gene expression) were obtained from the eQTLGen (https://eqtlgen.org/) consortium (v12) whole-blood cis-eQTL summary statistics (24). Outcome data (CRSwNP risk) were sourced from three independent CRSwNP GWAS datasets from the IEU OpenGWAS platform (IDs: ebi-a-GCST90018883, ukb-a-542, finn-b-J10_NASALPOLYP) (For more details, see **Supplementary Table 3**). SNPs significantly associated with the target gene (*P* < 5×10^-8^), independent (linkage disequilibrium r² < 0.001, distance > 10, 000 kb), were selected as IVs. The inverse-variance weighted (IVW) method was used as the primary analysis, supplemented by MR-Egger and weighted median methods. Horizontal pleiotropy, heterogeneity, and sensitivity were assessed using the MR-Egger intercept test, Cochran’s Q test, and the leave-one-out method, respectively.

### 2.11 qRT-PCR validation

Twenty-four CRSwNP tissues and sixteen healthy control subjects were collected with patient informed consent and ethical committee approval. The demographic data of the subjects are described in **Supplementary Table 5**. All patients had bilateral nasal polyps which partially or completely blocked the nasal cavity and underwent functional endoscopic sinus surgery. Inferior turbinate (IT) biopsies have been widely used as controls in CRSwNP studies (25) and were obtained from the patients with a septal deviation who were scheduled for septal surgery. None of the control subjects had upper respiratory infections or any type of rhinosinusitis.

Approval for this study was obtained from the institutional review board (IRB) of the Second Qilu Hospital of Shandong University, China. The review board protocol number is KYLL-2021(KJ)P-0242. Total RNA was extracted using TRIzol reagent and reverse-transcribed into cDNA. Amplification was performed using SYBR Green Master Mix on a real-time PCR system. GAPDH served as the internal reference gene. We calculated target gene expression levels relative to controls using the 2-ΔΔCt method. Wilcoxon rank-sum test was used to compare expression levels between groups. Primer sequences are listed in **Supplementary Table 6**.

### 2.12 Statistical analysis

All statistical analyses were conducted in R software (version 4.3.3). Before comparing two groups, normality of each group was assessed using the Shapiro-Wilk test, supplemented by visual inspection of Q-Q plots. If both groups passed normality and homogeneity of variance, the independent samples t-test was applied; otherwise, the Wilcoxon rank-sum test was used. Correlation analysis was performed using Spearman’s rank correlation. All statistical tests were two-sided, and a *P*-value < 0.05 was considered statistically significant.

## 3 Results

### 3.1 Identification and functional enrichment analysis of key metabolic reprogramming-related DEGs

**Figure 2:**
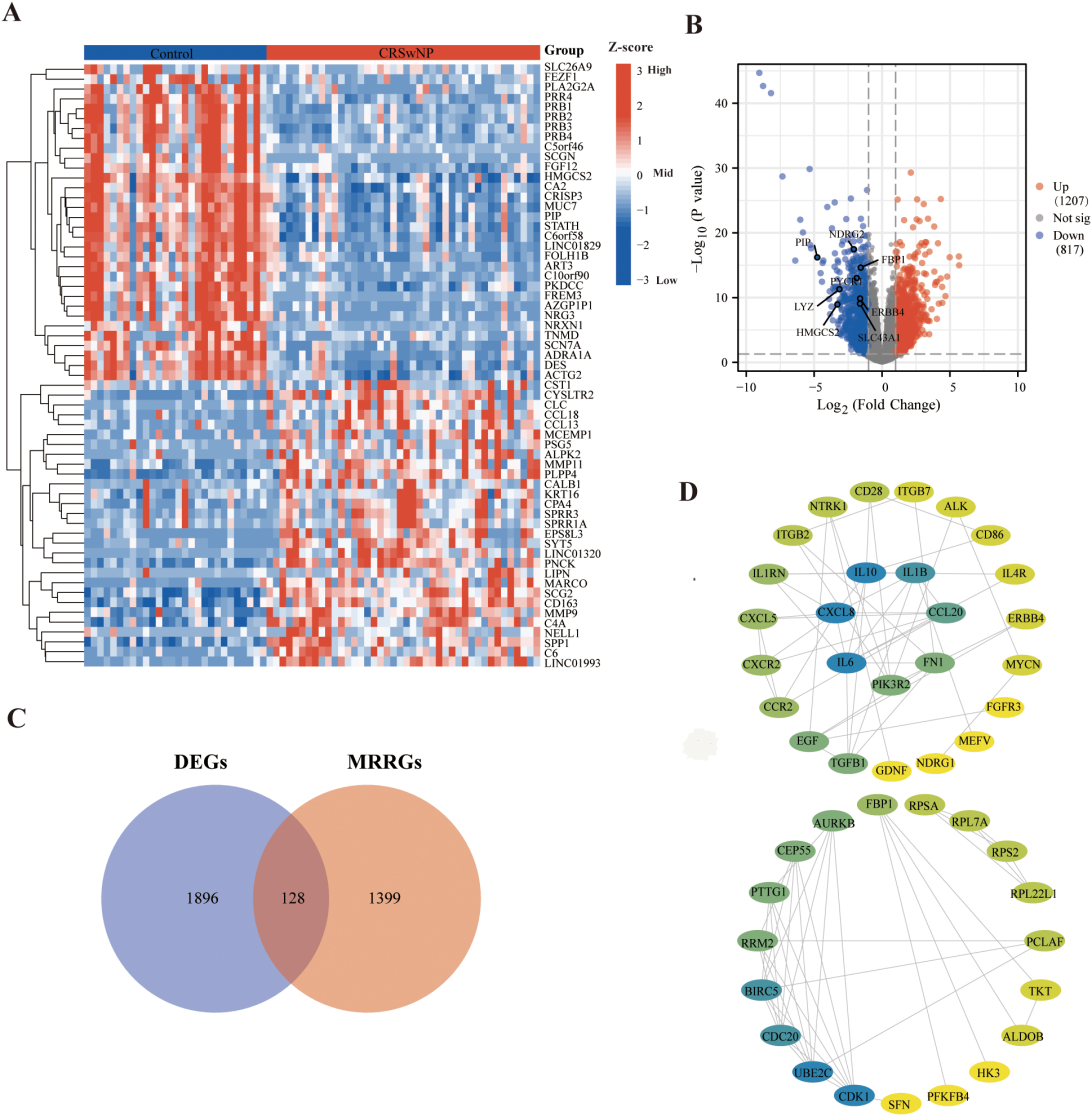
Identification of key metabolic reprogramming-related differentially expressed genes (DEGs). **(A)** Heatmap displaying the top 50 most significantly DEGs between CRSwNP and control groups. Rows (genes) were clustered using hierarchical clustering with Euclidean distance and complete linkage method. Expression values are row-normalized Z-scores. The color scale from blue to red represents low to high expression, as indicated on the color bar. Only genes meeting the significance threshold (P < 0.05) are shown. **(B)** Volcano plot depicting the DEGs identified between CRSwNP and control samples. 1207 Genes showing significant upregulation are marked in red, 817 downregulated genes in blue, and non-significant genes in gray. **(C)** Venn diagram representing the overlap between the set of DEGs (blue circle) and MRRGs (red circle). **(D)** Protein-protein interaction (PPI) network constructed from the 128 identified MR-DEGs. The network was constructed using the STRING database and visualized with Cytoscape (v3.9.1). Nodes (ovals) represent proteins, and edges indicate known or predicted functional associations. Node color: Nodes are colored using a continuous gradient from light yellow to dark blue, mapping the node’s degree (the number of direct interaction partners). Proteins with a higher degree (darker blue) represent central hubs in the network.

The GSE136825 dataset was obtained from GEO and analyzed with DESeq2 to identify CRSwNP-associated DEGs. Significance thresholds were set at |log2FC| > 1 and adjusted P-value < 0.05. A total of 2024 DEGs, including 1207 upregulated and 817 downregulated genes, were identified (**Figure 2B**). Volcano plots and a heatmap were then generated to visualize expression patterns. The heatmap (**Figure 2A**), which displays the top 50 most significant DEGs, clearly shows clustering separation between CRSwNP and control groups, confirming the reliability of the differential analysis.

**Figure 3:**
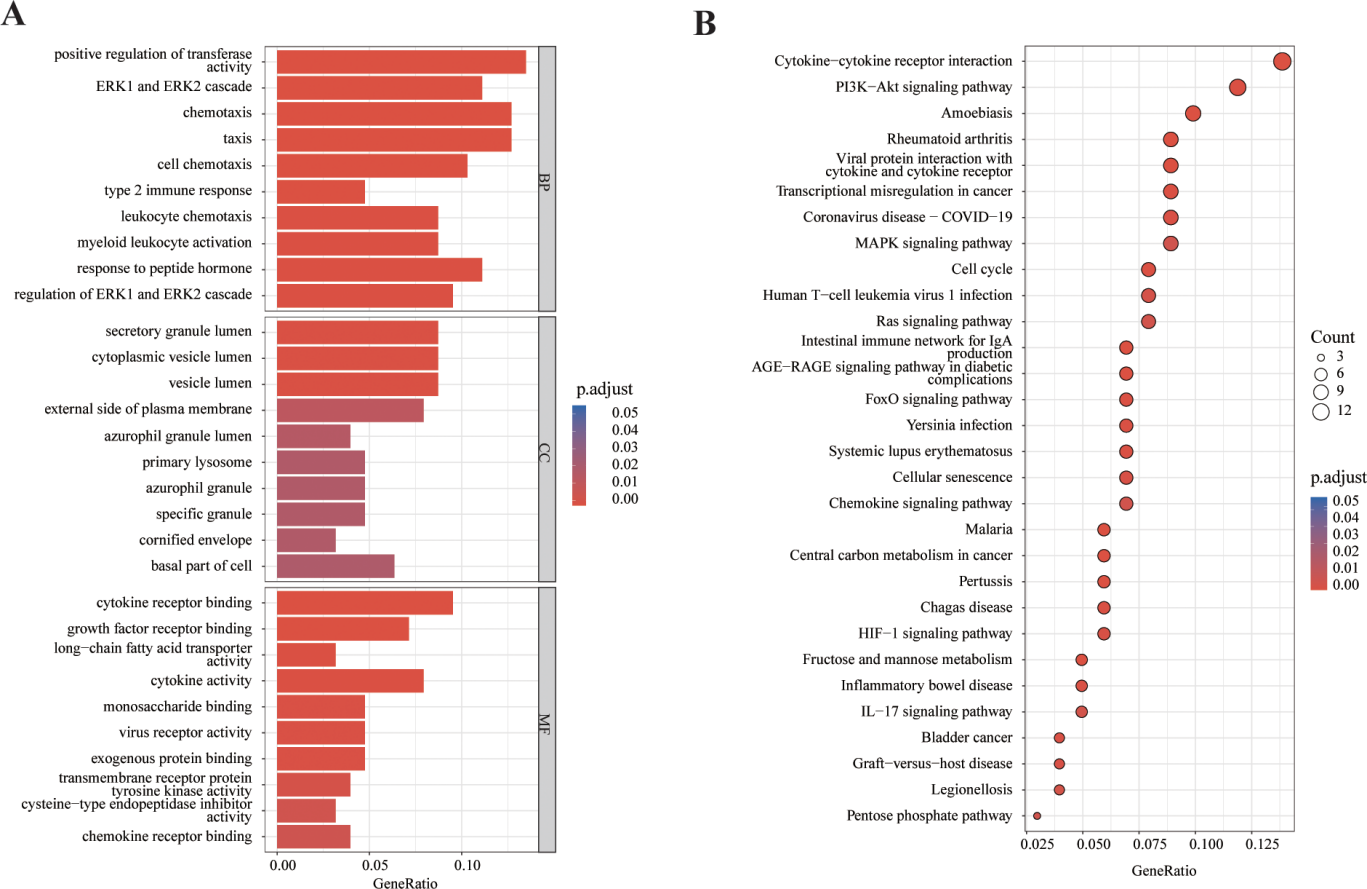
Functional enrichment analysis of key metabolic reprogramming-related differentially expressed genes (DEGs). **(A)** GO enrichment analysis highlighting significant terms in Biological Process (BP), Cellular Component (CC), and Molecular Function (MF). **(B)** KEGG pathway enrichment analysis of signaling pathways significantly enriched among the MR-DEGs.

To explore the connection between DEGs and metabolic reprogramming, we crossed the DEGs with a metabolic reprogramming-related gene set and ultimately discerned 128 overlapping MR-DEGs (**Figure 2C**). Nodes are colored using a continuous gradient from light yellow to dark blue, mapping the node’s degree (the number of direct interaction partners). These MR-DEGs exhibit relatively dense interaction relationships in the PPI network (**Figure 2D**). To elucidate the patient biological functions and pathways involving these MR-DEGs, we conducted GO and KEGG pathway analyses. Go analysis indicated significant enrichment for terms related to metabolic reprogramming, such as “Long-chain fatty acid transporter activity”, “monosaccharide binding”, “ERK1 and ERK2 cascade”, “regulation of ERK1 and ERK2 cascade” (**Figure 3A**). KEGG analysis revealed that the MR-DEGs were significantly enriched in pathways central to CRSwNP pathogenesis, including ‘Central carbon metabolism in cancer’, ‘Fructose and mannose metabolism’, ‘Pentose phosphate pathway’, and the ‘PI3K-Akt signaling pathway’ (**Figure 3B**). In summary, the key metabolic reprogramming-related DEGs identified provide new clues and perspectives for understanding the molecular mechanisms of metabolic reprogramming in CRSwNP development.

### 3.2 WGCNA of CRSwNP-related gene modules

**Figure 4:**
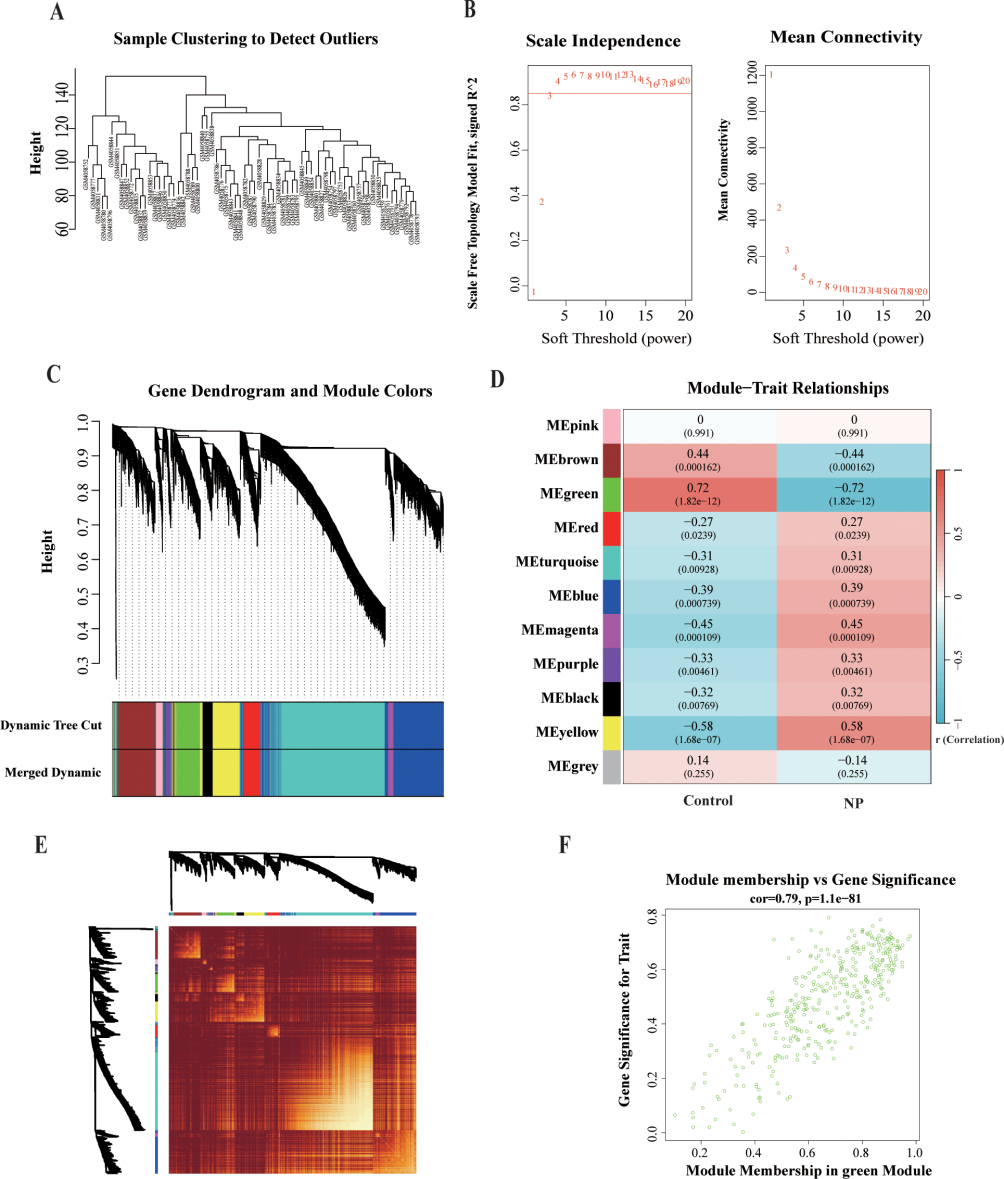
Construction of a weighted gene co-expression network (WGCNA). **(A)** Clustering dendrogram of samples used for outliers detection. **(B)** Analysis of scale-free topology assessment used to select optimal soft-thresholding power. **(C)** Clustering dendrogram of genes and the identified co-expression modules. **(D)** Module-trait correlation analysis illustrating the associations between gene modules and clinical characteristics. **(E)** Heatmap of the topological overlap matrix (TOM) among all genes, with module assignments indicated. **(F)** Scatter plot of module membership versus gene significance for CRSwNP in the green module.

Based on the GSE136825 dataset, we employed WGCNA to screen for modules highly correlated with the disease. Initial clustering of the 70 samples with the goodSamplesGenes algorithm confirmed no outliers, enabling subsequent module analysis (**Figure 4A**). Using a scale-free topology fit index >0.85, a soft-thresholding power of 4 was selected to create a network with relatively high average connectivity (**Figure 4B**). Dynamic tree cutting identified 11 gene modules which were further merged based on similarity (**Figure 4C**). Finally, a heatmap of the topological overlap matrix (TOM) was produced (**Figure 4E**).

Module-trait relationship analysis revealed that the green module (377 genes) exhibited the strongest negative correlation with the disease (*r* = -0.72, *P* = 1.8e-12), while the yellow module (485 genes) showed the strongest positive correlation (*r* = 0.58, *P* = 1.7e-07) (**Figure 4D**). This results suggest that the green module comprised genes that were likely exerting pathological influences in the disease process. Genes from this green module were selected as key candidates, on the basis of a significant positive correlation between module membership and gene significance. (*r* = 0.79, *P* = 1.1e-81; **Figure 4F**).

### 3.3 Mapping the dynamics of metabolic reprogramming to screen for diagnostic biomarkers in CRSwNP

Venn diagram analysis was performed on genes from the primary WGCNA module and the metabolic reprogramming-related gene set. This analysis identified 21 intersecting genes (**Supplementary Figure 3**).

**Figure 5:**
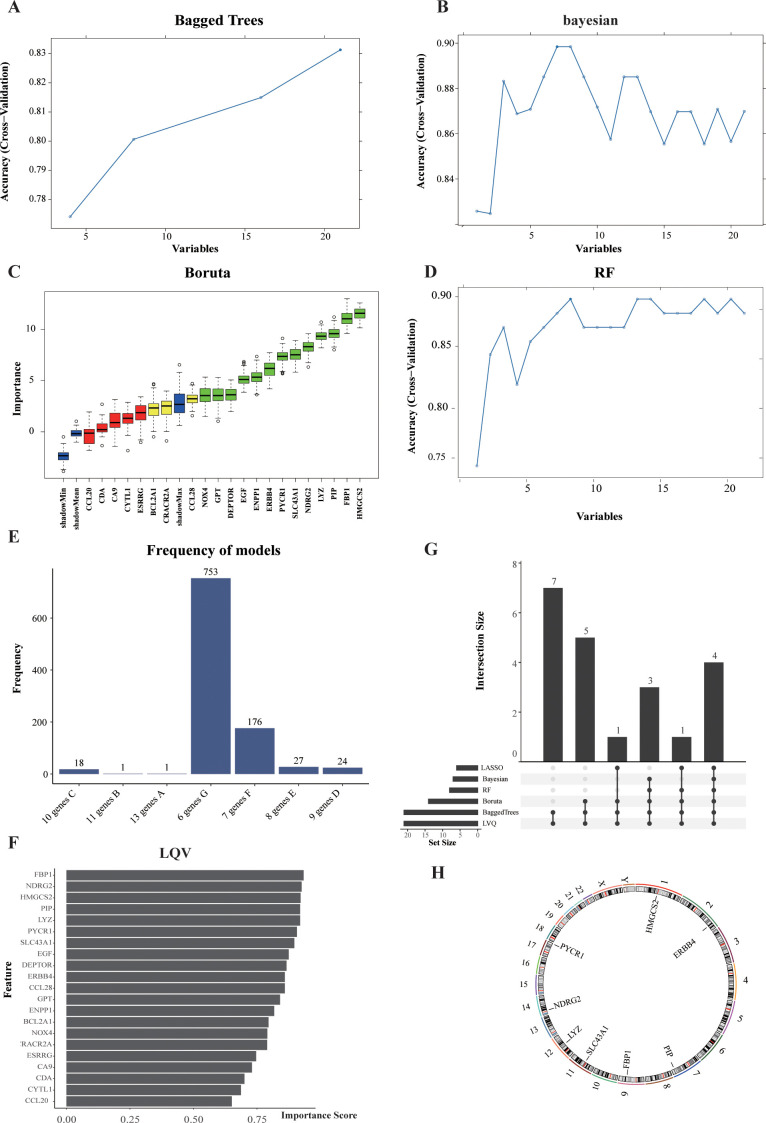
Identification of characteristic metabolic reprogramming-related biomarkers for CRSwNP using machine learning algorithms. **(A)** Distribution of variable importance scores and model accuracy for the Bagged Trees algorithm. **(B)** Accuracy distribution of the Bayesian algorithm across varying numbers of selected variables. **(C)** Feature selection results from the Boruta algorithm. Green and yellow boxes denote confirmed and tentative important features, respectively. **(D)** Accuracy distribution of the Random Forest (RF) algorithm with respect to the number of variables included. **(E)** Frequency of gene selection across 1, 000 runs of LASSO regression. Bar heights represent the occurrence frequency of each gene set. **(F)** Gene importance scores derived from the Learning Vector Quantization (LVQ). **(G)** Visualization of the upset plot of six different ML algorithms. Upper panel: Bar chart showing the intersection size (i.e., the number of shared genes) for each combination of ML algorithms. Lower panel: Matrix plot illustrating the composition of the specific algorithm combinations. Each row represents an algorithm, and black dots indicate that the algorithm is included in the corresponding feature intersection. The left axis indicates the total number of original features selected by each algorithm. **(H)** The chromosomal localization of these 8 hub-MRDEGs. The outer circle represents the human chromosomes (1-22, X and Y), with ribbons indicating the physical locations of specific genes (e.g., PYCR1, NDRG2, SLC39A1). The inner connections represent the interaction or association network among these genes.

Six ML approaches (Bagged Decision Tree, Naïve Bayes, Boruta, Random Forest, LASSO, and Learning LVQ) were applied to further identify key biomarkers associated with metabolic reprogramming in CRSwNP (**Figures 5A–F**). As different ML algorithms have different focuses, we integrated results from multiple algorithms to establish a more stable predictive model. Genes that were consistently identified by at least five algorithms were retained as the final set of hub-MRDEGs, resulting in eight genes (see **Figure 5G**): ERBB4, FBP1, HMGCS2, LYZ, NDRG2, PIP, PYCR1, and SLC43A1. The log2FC values and adjusted P-values of the eight hub-MRDEGs are presented in **Supplementary Table 1**. The detailed gene lists identified by each individual algorithm are provided in **Supplementary Table 2**.

Using the RCircos R package, we plotted the chromosomal localization of these 8 hub-MRDEGs (**Figure 5H**). The map shows HMGCS2 on chromosome 1, PIP on chromosome 7, ERBB4 on chromosome 2, SLC43A1 on chromosome 11, LYZ on chromosome 12, NDRG2 on chromosome 14, PYCR1 on chromosome 17, and FBP1 on chromosome 9.

### 3.4 Development of a diagnostic nomogram based on MR-derived biomarkers

**Figure 6:**
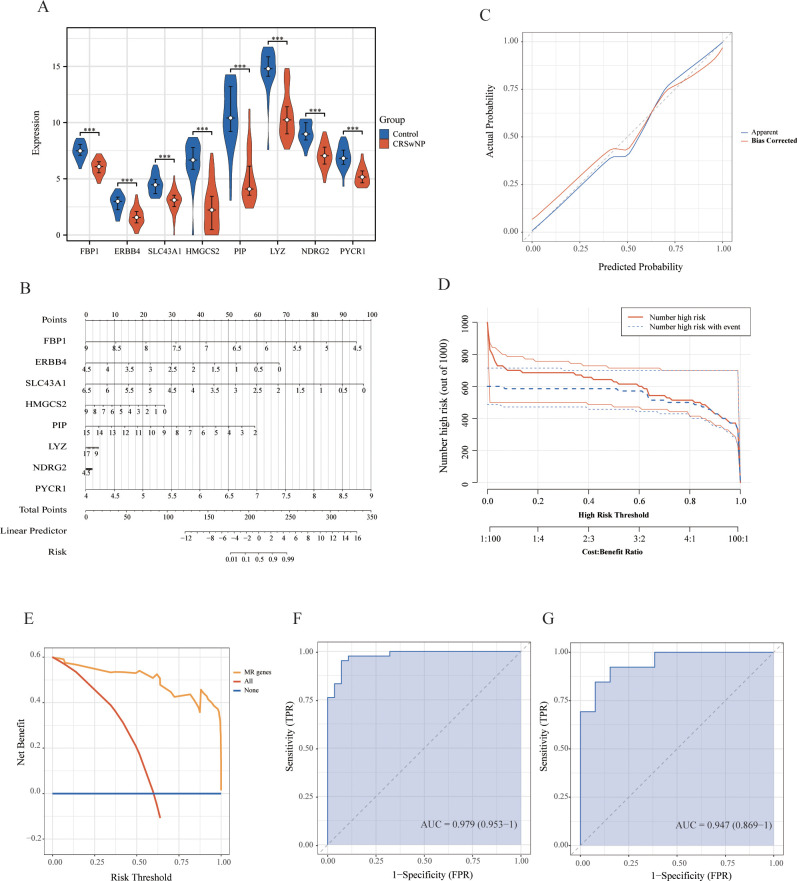
Development of a diagnostic nomogram based on MR-derived biomarkers. **(A)** The different expression profiles of FBP1, ERBB4, SLC43A1, HMGCS2, PIP, LYZ, NDRG2, and PYCR1 in CRSwNP tissues and controls were calculated by Wilcoxon rank-sum tests (median values with 25th and 75th percentiles are indicated by *scale bar*). **(B)** Nomogram model based on the eight genes. **(C)** Calibration curves of the CRSwNP risk models. **(D)** The clinical impact curve showed the clinical impact of the predictive model. **(E)** The DCA curve showed the benefits for patients of the nomogram model. **(F)** ROC curves of the eight biomarkers in the training set **(G)** ROC curves of the model in the dependent validation set.

To translate the discovered metabolic reprogramming signatures into a diagnostic tool, we evaluated their efficacy and constructed a predictive nomogram. Analysis within the training cohort revealed a significant downregulation of FBP1, ERBB4, SLC43A1, HMGCS2, PIP, LYZ, NDRG2, and PYCR1 in CRSwNP group compared to controls (**Figure 6A**).

A predictive nomogram was developed to estimate the risk of CRSwNP based on the expression profile of the eight key genes, with model construction and calibration performed using the “rms” package (**Figure 6B**). Calibration curve showed a good agreement between the predicted risk and actual risk (**Figure 6C**). In the range of 0.0–0.9 of threshold probabilities, DCA showed that the nomogram model has a greater net clinical benefit than either the “treat all” or “treat none” strategies (**Figure 6E**). Based on the DCA, we further plotted a clinical impact curve. For the range of threshold probabilities for high risk (0.6-1.0), the “Number high risk predicted” curve is close to our “Number high risk with event” (**Figure 6D**), indicating strong predictive capability of our model. Finally, ROC curves were plotted using the training set (GSE136825) and the independent validation set (merged GSE72713 and GSE179265) to evaluate performance of our model, with area under ROC curve (AUC = 0.979, AUC = 0.947) in both (**Figures 6F, G**), suggesting our model has strong discriminatory accuracy and potential for clinical use in CRSwNP.

### 3.5 Immune microenvironment profiling and correlation with MR-DEGs

**Figure 7:**
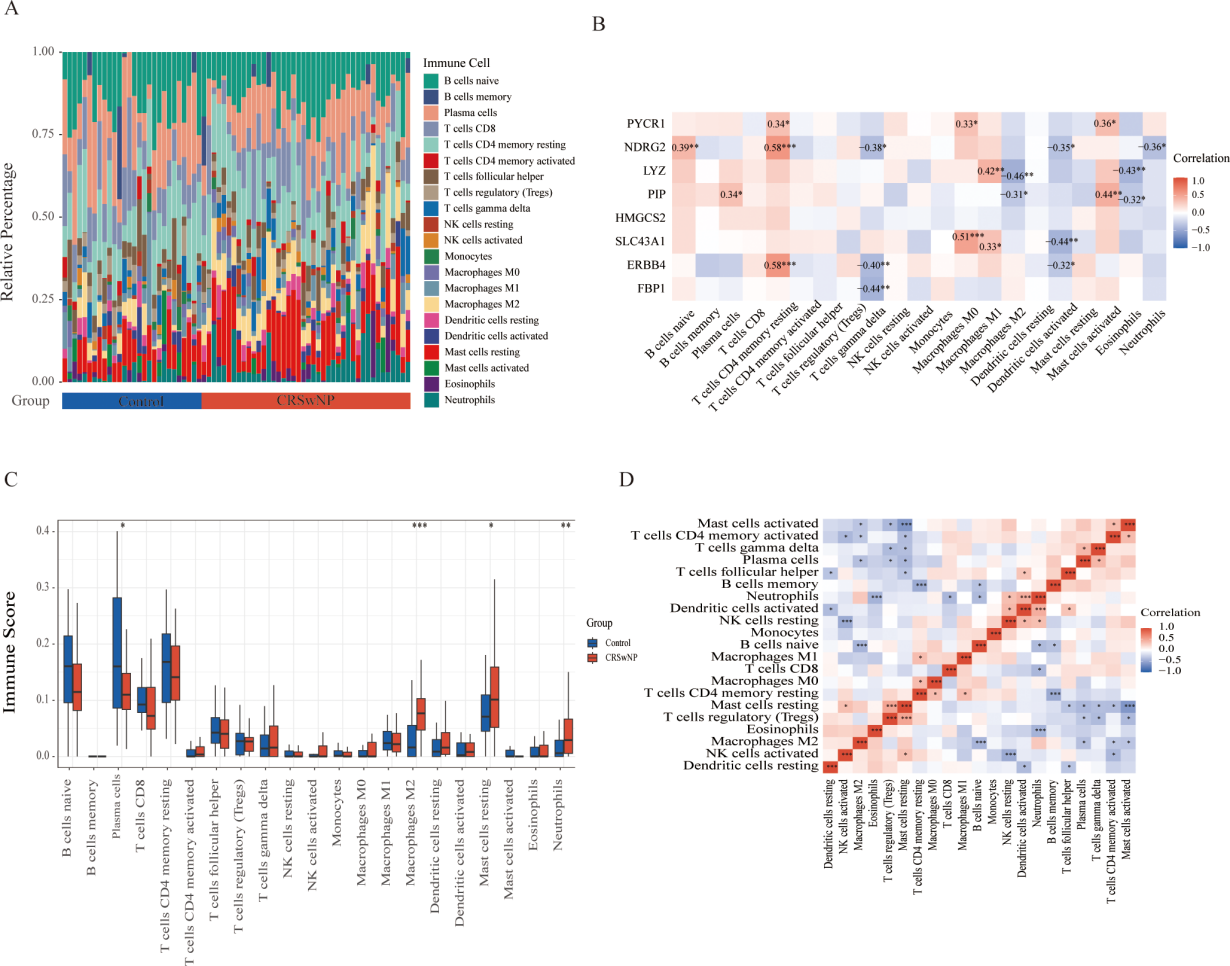
Immune microenvironment profiling and correlation with MR-DEGs. **(A)** CIBERSORT analysis of 22 immune cell types in control and CRSwNP groups. **(B)** Correlation heatmap between eight hub-MRDEGs and immune cell subtypes. **(C)** Differential immune cell infiltration between control (blue) and CRSwNP (red) groups. **(D)** Correlation heatmap among the 22 immune cell subtypes. Point size represents correlation coefficient magnitude; color indicates statistical significance (*P < 0.05; **P < 0.01; ***P < 0.001).

CIBERSORT analysis revealed significant alterations in the immune cell composition of CRSwNP. Specifically, the proportions of M2 macrophages, Neutrophils, and resting Mast cells were significantly increased (**Figure 7A**). Further assessment of correlations between subtypes was performed through a heat map. Resting Mast cells were strongly negatively correlated with activated Mast cells (correlation coefficient = -0.611), and memory B cells were negatively correlated with resting memory CD4+ T cells (-0.492) (**Figure 7D**). In particular, the proportions of plasma cells were lower in the CRSwNP group while the proportions of M2 Macrophages, resting Mast cells, and Neutrophils were significantly increased (**Figure 7C**).

Associations between the identified eight hub-MRDEGs (ERBB4, FBP1, HMGCS2, LYZ, NDRG2, PIP, PYCR1, SLC43A1) and immune cell features were statistically evaluated using Spearman correlation analysis (**Figure 7B**). A group of genes, including FBP1, ERBB4, NDRG2, and PYCR1, showed a consistent negative association with effector immune cells such as gamma delta T cells and activated dendritic cells. Notably, ERBB4 and NDRG2 were also positively correlated with resting memory CD4+ T cells, suggesting a potential role in regulating adaptive immune responses. Conversely, another gene set comprising SLC43A1, PIP, and LYZ was primarily linked to the composition of myeloid cells. These genes exhibited significant positive correlations with various macrophage subsets (M0 and/or M1) but negative associations with other innate immune populations like activated dendritic cells, eosinophils, or M2 macrophages. Collectively, these findings suggest that the identified genes may critically shape the CRSwNP immune microenvironment by differentially influencing the infiltration and balance of major lymphocyte and myeloid cell populations.

### 3.6 Single-cell transcriptomic analysis of nasal polyps

**Figure 8:**
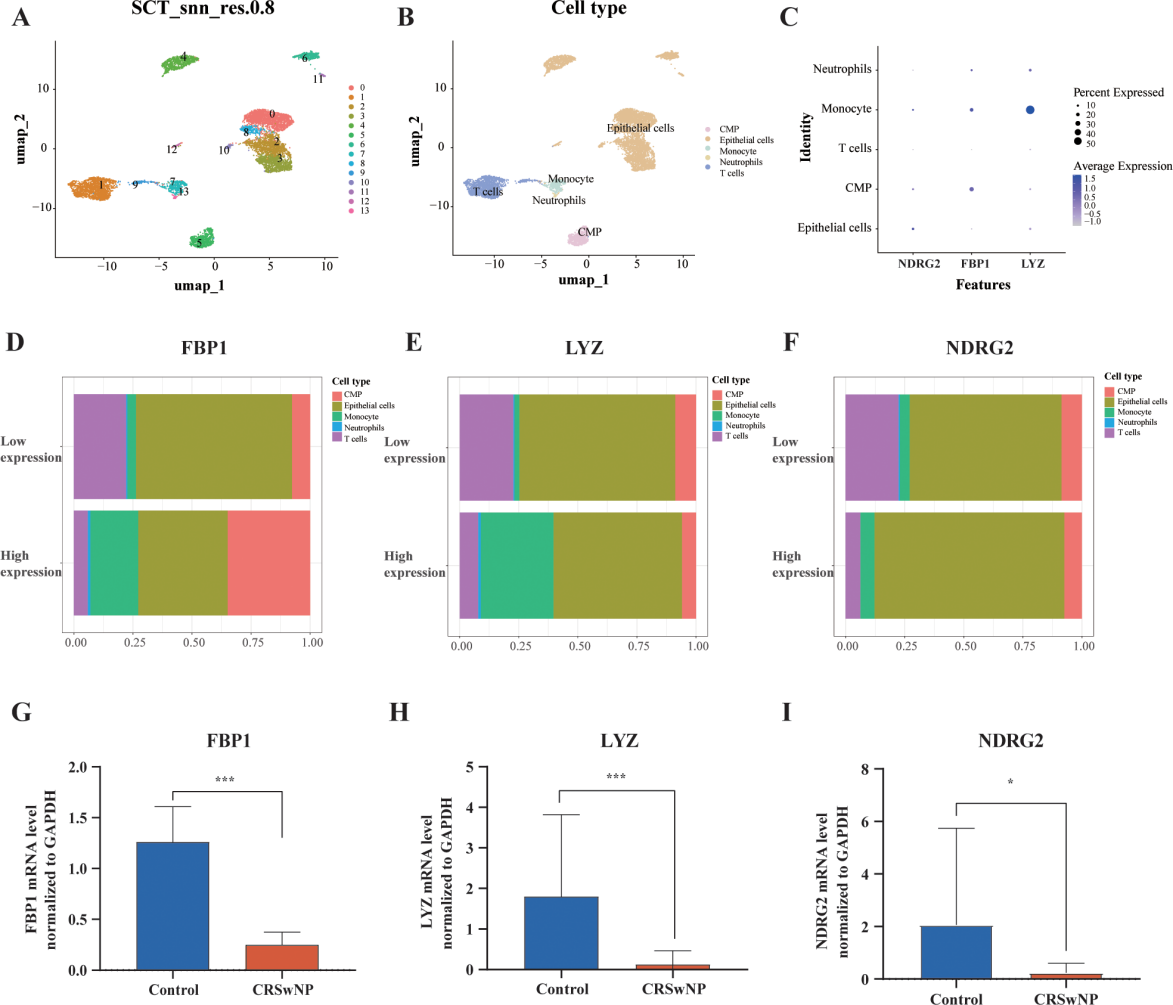
Single-cell transcriptomic analysis of nasal polyps. **(A)** After rigorous quality control (filtering low-quality cells and doublets), a total of 8, 046 cells were included in the UMAP analysis. Based on transcriptomic features, cell clustering identified 13 clusters, which are shown in different colors. **(B)** UMAP plot colored by cell type annotation (CMPs, epithelial cells, monocytes, neutrophils, T cells). **(C)** Bubble plot of hub-MRDEGs expression across cell types. **(D)** Cell type composition in groups stratified by high versus low FBP1 expression. **(E)** Cell type composition in groups stratified by high versus low LYZ expression. **(F)** Cell type composition in groups stratified by high versus low NDRG2 expression. FBP1 **(G)**, LYZ **(H)** and NDRG2 **(I)** expression in control and CRSwNP groups (*P < 0.05; **P < 0.01; ***P < 0.001).

scRNA-seq data from CRSwNP (GSE202100) were analyzed. UMAP visualization of these data clustered all cells into 13 clusters (**Figure 8A**). Using distinct features, these were assigned to five main cell types: CMPs, Epithelial cells, Monocytes, Neutrophils and T cells (**Figure 8B**). The expression patterns of key marker genes across these cell types are shown in a dot plot (**Figure 8C**). This analysis suggested that FBP1 expression was high in Monocytes and CMP, and indicates a likely context specific functional role for the gene. LYZ was enriched in Monocytes and Neutrophils, while NDRG2 expression was higher in Epithelial cells and Monocytes.

Cells were stratified based on FBP1 expression levels. The low-FBP1 group was predominantly comprised of CMPs, epithelial cells, and monocytes (**Figure 8D**). Similarly, stratification by LYZ expression revealed that high-LYZ cells were mainly epithelial cells and monocytes, whereas low-LYZ cells were primarily epithelial cells and T cells (**Figure 8E**). Finally, we found the NDRG2 low-expression group would be predominantly of epithelial cells and T cells (**Figure 8F**).

### 3.7 MR analysis of metabolic reprogramming-related gene expression

**Figure 9:**
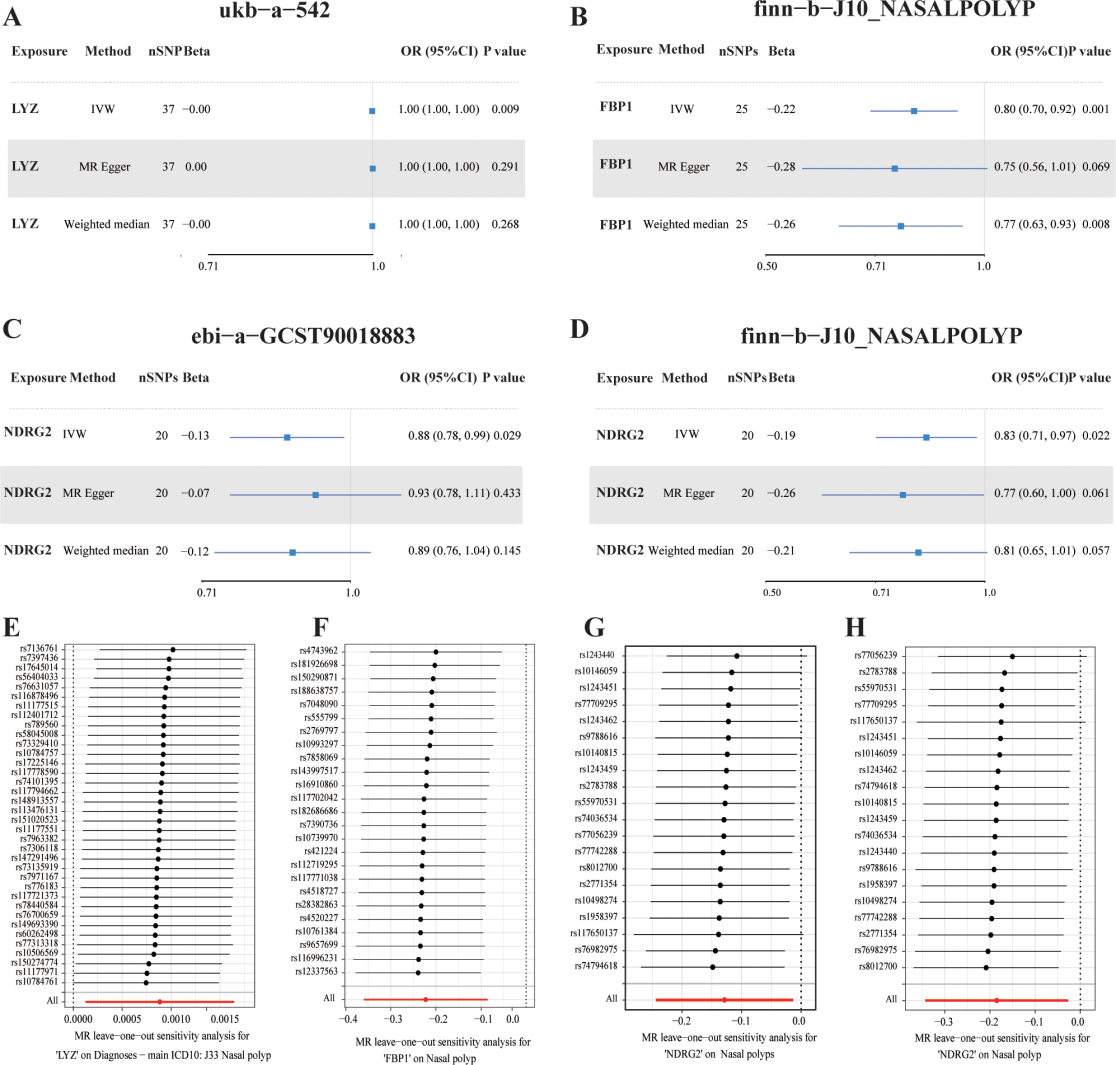
Mendelian randomization analysis of metabolic reprogramming related gene expression. Mendelian randomization analysis of gene expression on CRSwNP risk. Forest plots for LYZ, FBP1, and NDRG2, respectively, showing odds ratios (ORs) and 95% confidence intervals from multiple MR methods **(A–D)**. Leave-one-out sensitivity analysis for LYZ, FBP1, and NDRG2 (two panels), respectively **(E–H)**.

We performed a two-sample Mendelian randomization study for identifying any potential causal relationships between the expression of the eight hub genes and the risk of CRSwNP. For five genes (ERBB4, HMGCS2, PIP, PYCR1, and SLC43A1), we were unable to identify a sufficient number of valid instrumental variables meeting the required criteria. Using weak or insufficient genetic variants in Mendelian randomization would introduce bias and yield unreliable causal estimates; therefore, these genes were excluded from the analysis. Consequently, we focused on and successfully validated the key genes FBP1, LYZ, and NDRG2. The Inverse-Variance Weighted (IVW) method indicated that genetically predicted higher expression of LYZ and NDRG2 was significantly linked to lower CRSwNP risk (OR < 1, *P* < 0.05), while MR-Egger and Weighted Median methods showed similar trends without reaching statistical significance (**Figure 9A**). The IVW and Weighted Median methods showed that genetically predicted higher FBP1 expression was significantly associated with a lower risk of CRSwNP (OR < 1, *P* < 0.05) while the direction of association from MR-Egger was consistent, it did not achieve statistical significance (**Figure 9A**). It is worth noting that the OR value for LYZ, while statistically significant, indicates a clinically negligible effect size.

Leave-one-out sensitivity analysis (**Figures 9E–H**) confirmed the stability of the effect estimates, with no single SNP unduly influencing the results. Further sensitivity analysis revealed no evidence of significant horizontal pleiotropy or heterogeneity (**Supplementary Table 4**).

The inverse relationship between key genes (FBP1, LYZ, and NDRG2) expression and CRSwNP risk was further reinforced by scatter plots integrating the collective results from multiple MR methods (**Supplementary Figure 1**). Together, these MR results support a potential causal role for reduced expression of FBP1, LYZ, and NDRG2 in increasing CRSwNP risk.

### 3.8 qRT-PCR validation

To confirm the involvement of metabolic reprogramming-related genes in CRSwNP, qRT-PCR analysis was performed on CRSwNP tissues (n=24) and normal tissues (n=16) (**Supplementary Table 5**). Expression levels of FBP1, LYZ, and NDRG2 were markedly downregulated in the CRSwNP group relative to controls (*P* < 0.01, **Figures 8G–I**). These results experimentally corroborate the findings from our bioinformatics and MR analyses. The expression levels of ERBB4, HMGCS2, PIP, PYCR1, and SLC43A1 in control and CRSwNP groups were shown in **Supplementary Figure 2**.

## 4 Discussion

Chronic rhinosinusitis with nasal polyps (CRSwNP) is typified by chronic inflammation and polypoid change within the sinonasal mucosa. Patients suffer from refractory symptoms and disease recurrence, leading to poor quality of life and ongoing management dilemmas. Traditionally research has focused on Th2-type immune inflammation and aberrant functional activity of the relevant immune cell types. Recent studies suggest that metabolic reprogramming-the rewiring of central metabolic pathway to meet bioenergetic and biosynthetic needs-is a fundamental driver of many chronic inflammatory diseases (26–28). This insight offers a new avenues for investigating the mechanisms underlying persistent inflammation in CRSwNP.

In CRSwNP, immunometabolic reprogramming is so closely linked to immune cell function, directly influencing therapeutic responses. For instance, CD45RO+ inflammatory ILC2 cells acquire glucocorticoid resistance through metabolically activated detoxification (26). Dendritic cells, monocytes, macrophages and T cells all undergo metabolic reprogramming. These metabolic changes not only provide energy but dictate how immune cells are activated, differentiated and effector functions, and therefore program the entire inflammatory response (28, 29).

Particularly remarkable are the dramatic metabolic changes occurring in monocytes and macrophages within the chronic inflammation. Leucine uptake through the amino acid transporter SLC7A5 can, after immune activation, activate, via mTORC1, glycolytic reprogramming, which in turn drives the production of pro-inflammatory cytokines such as IL-1β by monocytes and macrophages (29). Furthermore, macrophages regulate their polarization, and inflammatory mediators production by regulating glycolysis, fatty acid oxidation and amino acid metabolism, mechanisms that contribute to the maintenance of chronic inflammation (30). This crosstalk between metabolism and immunity gives a new perspective to understanding the persistence and recurrence of inflammation in CRSwNP.

Genetic and epigenetic studies have highlighted the significance of metabolic regulation in CRSwNP pathogenesis. In particular, a systematic evaluation demonstrated that over 150 gene variants associated with nasal polyps were significantly enriched in inflammatory response, immune regulation and cell cycle and notably, extracellular matrix remodeling (31). Concomitantly, dysregulated non-coding RNAs, such as microRNAs, modulate the interplay between inflammation and metabolism through specific signaling pathways (32). In this regard, metabolic regulation of gene expression through epigenetic features such as DNA methylation and histone modifications contributes to CRSwNP development.

In this study, we examined the important role of metabolic reprogramming in CRSwNP using an integrative bioinformatics strategy. We first collected a set of genes related to metabolic reprogramming in the Gene Cards database and integrated it with CRSwNP-related datasets from GEO. Following integration and differential expression analysis, we identified 128 significantly dysregulated genes. Subsequently, eight core metabolic reprogramming-related diagnostic markers: ERBB4, FBP1, HMGCS2, LYZ, NDRG2, PIP, PYCR1, and SLC43A1 were screened by WGCNA and machine learning algorithms after that to build an efficient diagnostic model.

Multiple studies have demonstrated that various biomarkers are closely associated with the pathogenesis, disease severity, and postoperative recurrence of CRSwNP. Using WGCNA and machine learning methods, researchers successfully identified 18 M2 macrophage−related hub genes (e.g., AIF1, C1QA, CD163), suggesting their key roles in the pathogenesis of CRSwNP (33). Another study constructed an M2 macrophage−related diagnostic model containing HMOX1, ALOX5, F13A1, and ITGB2, achieving AUCs of 0.980 in the training cohort and 0.895 in the test cohort (34). Metabolomic biomarkers (serum citrulline, linoleic acid, adenosine) combined with immune markers showed good predictive value in distinguishing eosinophilic from non−eosinophilic CRSwNP (35). Regarding recurrence risk prediction, a five−molecule signature (S1PR5, MerTK, MFGE8, CD300a, PPARδ) predicted CRSwNP recurrence with an AUC of 0.867 (36). A multiplex analysis of 71 cytokines/chemokines coupled with machine learning identified IL−21 and MIP−1δ as associated with olfactory dysfunction in CRSwNP patients (37).

Compared with previously reported molecular markers for NPs, our model integrates eight metabolic reprogramming−related genes and captures the complex interplay between metabolism and immunity. Methodologically, we combined six complementary machine learning algorithms with WGCNA, effectively reducing overfitting and enhancing generalizability. Furthermore, the introduction of two−sample Mendelian randomization provides causal inference support that is generally lacking in observational biomarker studies. Ultimately, our model achieved AUCs of 0.979 in the training set and 0.947 in the external validation set. These features collectively underscore the robustness and translational potential of our metabolic reprogramming−based model.

In addition, we explored the infiltration levels of the 22 immune cell types using the CIBERSORT algorithm. Results revealed that relative to controls, the CRSwNP group displayed a markedly lower proportion of plasma cells, coupled with significantly higher levels of infiltration by M2 macrophages, resting mast cells, and neutrophils overall. Expression levels of the eight core markers exhibited significant correlations with immune cell infiltration, highlighting their importance in CRSwNP microenvironment.

Through a Multidimensional Transcriptome Integration strategy, we pinpointed FBP1, LYZ, and NDRG2 as pivotal proteins involved in metabolic reprogramming in CRSwNP. Owing to their central significance, these genes were assessed as prospective diagnostic biomarkers and therapeutic targets. Their association with CRSwNP risk was robustly validated through multiple complementary analytical methods. To further investigate the possible causal association, Mendelian randomization (MR) analysis found that occurrence of CRSwNP was inversely associated with genetically predicted expression of FBP1, LYZ, and NDRG2. Compared to purely observational studies, this MR analysis provides stronger evidence for a causal regulatory role in the development of CRSwNP and hence offers direct mechanistic insights.

Further scrutiny of the functions of these hub proteins provides insight into their likely mechanisms in our system. As a rate-limiting enzyme of gluconeogenesis, Fructose-1, 6-bisphosphatase 1 (FBP1) plays a central role in maintaining metabolic homeostasis in the cell, but also regulates cell energy, proliferation and apoptosis. In our study, we found that FBP1 expression was lower in CRSwNP tissues than in control tissues as it showed in tumors and other chronic inflammatory diseases. This FBP1 deficiency drives glycolysis rate, inflammatory mediators and cell proliferation (38, 39). Downregulation of FBP1 may thus reprogram the metabolism of local immune cells and promote an inflammatory microenvironment conducive to tissue remodeling (40, 41). Thus, reduced FBP1 in CRSwNP likely represents not a passive consequence but an active driver of the disease process.

Another important antimicrobial protein is lysozyme (LYZ), playing a critical role in innate immunity by hydrolyzing peptidoglycan in bacterial cell walls (42). Our findings reveal that the expression of LYZ is downregulated in CRSwNP tissues. This may compromise local antibacterial defenses and the integrity of mucosal barriers, leading to the emergence of a microenvironment permissive to bacterial colonization and sustained inflammation (43, 44). Thus, abnormal LYZ expression is, simultaneously, a manifestation of impaired immune defense in CRSwNP, and a contributor to the disease process by disrupting mucosal homeostasis.

Finally, NDRG2 is a multifunctional stress-responsive gene regulating cell cycle, apoptosis and homeostasis. In inflammatory conditions such as rheumatoid arthritis, low NDRG2 levels promote abnormal proliferation of fibroblast-like synoviocytes, contributing to the inflammatory process (45). Here, we confirm low expression of NDRG2 in CRSwNP tissues, consistent with evidence of cell cycle dysregulation and inhibition of apoptosis (46). Thus, we propose that downregulation of NDRG2 promotes abnormal hyperplasia and inflammation in CRSwNP tissues. Overall, the molecular changes we describe implicate a metabolic derangement as a potential core feature driving CRSwNP.

Using single-cell RNA sequencing, we further investigated the functional states of key cell types in nasal polyps tissues. This analysis identified epithelial cells, monocytes, and common myeloid progenitors (CMPs) as the major metabolic reprogramming-related cell populations. In CRSwNP patients, the nasal epithelial barrier function is severely compromised with tight junction proteins reduced, presumably increasing the epithelium’s permeability further promoting inflammatory cell migration and penetration of antigens (47, 48). As progenitors of the myeloid cells, CMPs may be involved in the immunopathology by supplying and potentially influencing the metabolic state of downstream effector cells. Monocytes, as critical inflammatory modulating cells, may expand locally in CRSwNP, secreting various cytokines and modulating the immune microenvironment through cytokine networks (49, 50).

Beyond conventional anti−inflammatory therapies, this study proposes a new metabolic−targeting perspective for chronic rhinosinusitis with nasal polyps (CRSwNP). FBP1, a rate−limiting gluconeogenic enzyme, holds promise as a candidate target. However, clinical translation of FBP1 agonists requires overcoming challenges related to safety and delivery, such as employing local administration (e.g., nasal sprays or nanocarriers) to avoid systemic metabolic side effects. Furthermore, patient stratification based on the expression signature of these eight genes facilitates precision medicine and may establish metabolically−driven targeted therapy as a new cornerstone for CRSwNP treatment.

Although this study has identified several promising biomarkers, some findings (particularly those from scRNA−seq and qRT−PCR analyses) are based on a limited single−center cohort, and strict inclusion/exclusion criteria have resulted in a relatively small number of eligible specimens. Future multicenter, large−scale prospective cohort studies are needed to validate the expression levels and diagnostic performance of key molecules (FBP1, LYZ, NDRG2) in independent external cohorts. Furthermore, the LM22 signature matrix used for immune infiltration analysis is derived from peripheral blood cell expression profiles and may not fully capture the true phenotypic characteristics of tissue−resident immune cells in the nasal mucosa. Constructing a tissue−specific deconvolution reference matrix requires purified cell populations or rigorously sorted single−cell transcriptomic data from the same tissue type. Future studies with larger sample sizes and matched single−cell data are warranted to improve the accuracy of immune infiltration estimation. In addition, the PPI network analysis assessed node degree but lacked systematic topological characterization (e.g., centrality, modularity), and the two observed gene clusters were not separately analyzed for their distinct biological meanings. Therefore, the inference of a cooperative functional module remains preliminary and requires future experimental and computational validation.

## 5 Conclusions

Our research confirmed the characteristics of metabolic reprogramming in CRSwNP and identified molecules like FBP1, LYZ, and NDRG2, along with their preliminary validation of exposure–disease associations using Mendelian randomization. These results not only offer new insights into CRSwNP pathogenesis, but also lay a foundation for future efforts in personalized treatment and risk prediction. Future studies are encouraged to focus on functional validation of these hub genes and systematically explore their utilization for clinical diagnosis and targeted therapy.

## Data Availability

The datasets presented in this study can be found in online repositories. The names of the repository/repositories and accession number(s) can be found in the article/**Supplementary Material**.
