# Supplementary material for: Diagnostic value and immune microenvironment regulatory network of metabolic reprogramming in chronic rhinosinusitis with nasal polyps identified by multidimensional transcriptome integration and machine learning
Source: Front Immunol. 2026 May 25;17:1808799. doi: 10.3389/fimmu.2026.1808799 (PMC13243060; doi:10.3389/fimmu.2026.1808799)
Supplement: Supplementary file 1 [file DataSheet1.pdf]

Supplementary Material

Table S1. The log2FC values and adjusted P-values of the eight hub-MRDEGs.

Gene	log2FoldChange	P adj
HMGCS2	-3.287483781	$3.54X10^{-08}$
ERBB4	-1.615873306	$5.78X10^{-09}$
PIP	-4.76953796	$1.42X10^{-14}$
FBP1	-1.573650609	$3.47X10^{-13}$
SLC43A1	-1.627122543	$2.82X10^{-08}$
LYZ	-3.135467048	$3.15X10^{-10}$
NDRG2	-2.077459728	$1.11X10^{-15}$
PYCR1	-1.868306525	$9.59X10^{-12}$

Table S2. The list of genes identified by each individual algorithm

Machine Learning	Genes	No. genes
Bagged Decision Tree	HMGCS2, LYZ, FBP1, PIP, NDRG2, PYCR1, SLC43A1, ERBB4, EGF, ENPP1, BCL2A1, DEPTOR, NOX4, GPT, CRACR2A, CYTL1, CCL20, ESRRG, CCL28, CDA, CA9	21
Naïve Bayes	FBP1, NDRG2, HMGCS2, PIP, LYZ, PYCR1, SLC43A1	7
Boruta	NOX4, ENPP1, GPT, FBP1, ERBB4, SLC43A1, PYCR1, DEPTOR, NDRG2, CCL28, EGF, LYZ, HMGCS2, PIP	14
Random Forest	FBP1, HMGCS2, PIP, LYZ, NDRG2, ERBB4, SLC43A1, PYCR1	8
LASSO	ENPP1, ERBB4, FBP1, HMGCS2, PIP, SLC43A1	6
LVQ	NOX4, CYTL1, CA9, BCL2A1, CCL20, CDA,ESRRG, CRACR2A, ENPP1, GPT, FBP1,ERBB4, SLC43A1, PYCR1, DEPTOR, NDRG2, CCL28, EGF, LYZ, HMGCS2, PIP	21

Table S3. Information of GWAS summary statistics for MR analyses

GWAS ID	Year	phenotype	Cases	Controls	population
ebi-a-GCST90018883	2021	Nasal polyps	5093	444966	European
ukb-a-542	2017	Diagnoses - main ICD10: J34 Other disorders of nose and nasal sinuses	3433	333766	European
finn-b-J10_NASALPOLYP	2021	Nasal polyp	3236	167849	European

Table S4. Pleiotropy and heterogeneity test of the MR analysis.

id.outcome	Gene	Pleiotropy		Heterogeneity			
		**Egger intercept**	***P*-value**	**IVW Q**	***P*-value**	**MR Egger Q**	***P*-value**
finn-b-J10_NASALPOLYP	FBP1	0.0079	0.64	19.86	0.70	19.64	0.66
ukb-a-542	LYZ	0.00012	0.21	37.17	0.36	35.49	0.39
ebi-a-GCST90018883	NDRG2	-0.0086	0.39	14.45	0.76	13.68	0.75
finn-b-J10_NASALPOLYP	NDRG2	0.0098	0.47	12.30	0.87	11.77	0.86

Cochran’s Q statistic indicated the absence of heterogeneity.No significant pleiotropy as observed in these analyses ($P > 0.05$).

Table S5. Basic Information of Patients for qRT-PCR

	Control (n=16)	NP (n=24)	*P* value
Gender (M/F)	14/2	19/5	ns
Age (yr)	26.5 (18.75, 35.25)	33.5 (31.0, 55.75)	ns
Atopy (n)	0	4	
Asthma (n)	0	1	
Smoking (n)	3	7	ns

The difference in gender, age and smoking status between NPs patient group and control group were also stratified and compared using the Wilcoxon rank-sum test. A p value of less than 0.05 was considered statistically significant. (ns, not significant).

Table S6. Primer sequences used in this study.

Genes		Primers (5′–3′)
ERBB4	F	GTCCAGCCCAGCGATTCTC
	R	AGAGCCACTAACACGTAGCCT
FBP1	F	CGCGCACCTCTATGGCATT
	R	TTCTTCTGACACGAGAACACAC
HMGCS2	F	GACTCCAGTGAAGCGCATTCT
	R	CTGGGAAGTAGACCTCCAGG
LYZ	F	CTTGTCCTCCTTTCTGTTACGG
	R	CCCCTGTAGCCATCCATTCC
NDRG2	F	AGACTCACTCTGTGGAGACAC
	R	CGTGGTAGGTAAGGATCGCTG
PIP	F	GCCAACAAAGCTCAGGACAAC
	R	CGTCATTTGGACGTACTGACTTG
PYCR1	F	TGGCTGCCCACAAGATAATGG
	R	CGTGACGGCATCAATCAGGT
SLC43A1	F	GGACGTGGAAGCTCTGTCTC
	R	GCAGCGTGAGTGAAGTGAAC
GAPDH	F	GAGTCAACGGATTTGGTCGT
	R	GACAAGCTTCCCGTTCTCAG

Figure legends

Figure S1 Mendelian randomization analysis of MR-DEGs.

Scatter plots illustrate the inverse relationship of FBP1 (A), LYZ (B), and NDRG2 (C-D) with NPs.

Figure S2. qRT-PCR validation.

Comparison of gene expression in ERBB4 (A), HMGCS2 (B), PIP (C), SLC43A1(D) and PYCR1 (E) between NPs and control group (*$P < 0.05$, **$P < 0.01$,***$P < 0.001$, ns=not significant).

Figure S3 Venn diagram.

The intersection of the WGCNA green module (blue circle, containing 377 genes), which showed the strongest correlation with nasal polyp status, and the 128 previously identified metabolic reprogramming-related DEGs (red circle) yielded 21 core candidate genes.
