# Supplementary figures and images for: Diagnostic value and immune microenvironment regulatory network of metabolic reprogramming in chronic rhinosinusitis with nasal polyps identified by multidimensional transcriptome integration and machine learning
Source: Front Immunol. 2026 May 25;17:1808799. doi: 10.3389/fimmu.2026.1808799 (PMC13243060; doi:10.3389/fimmu.2026.1808799)

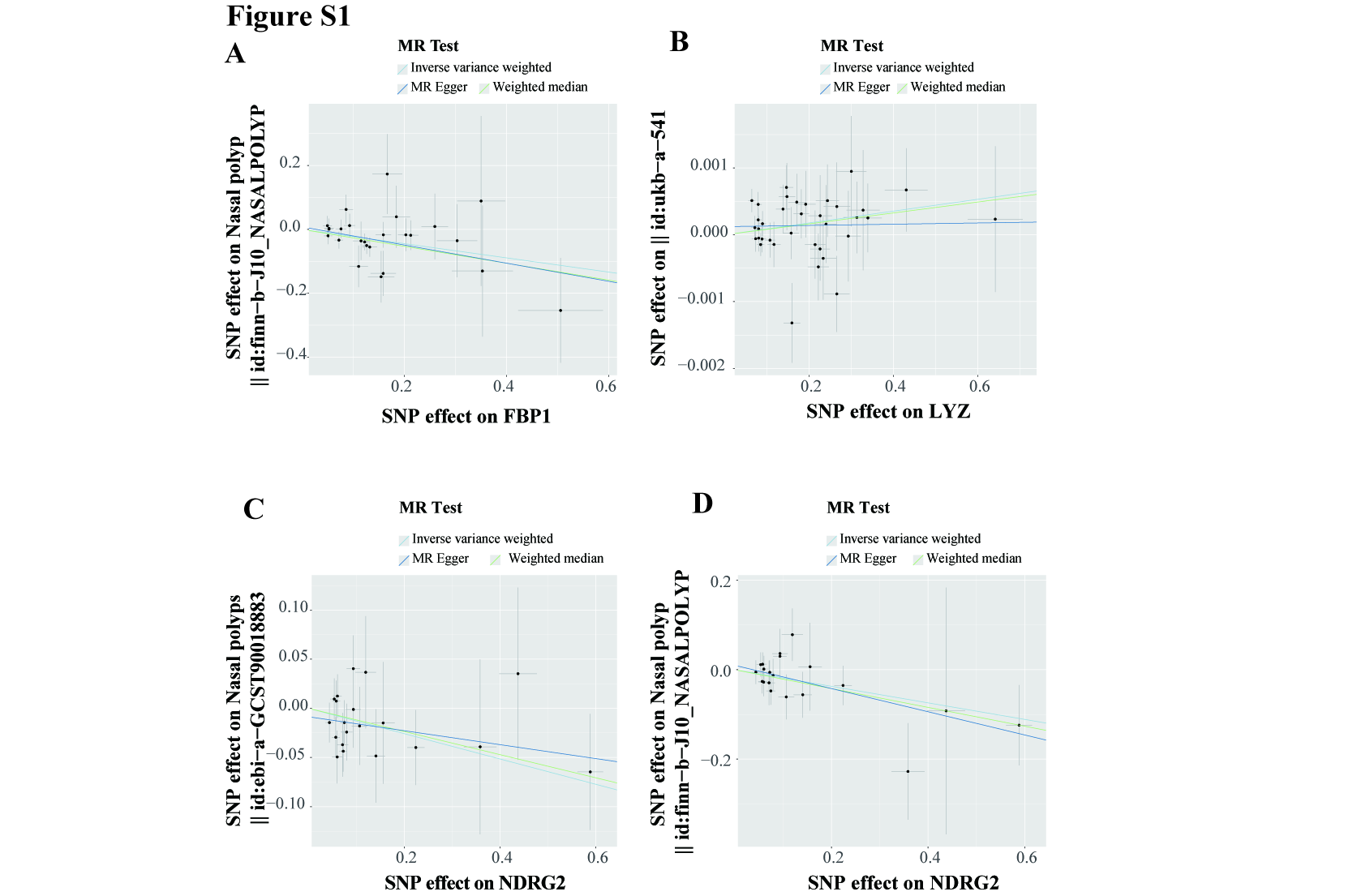

Supplement: Supplementary file 2 [file Image1.tif]

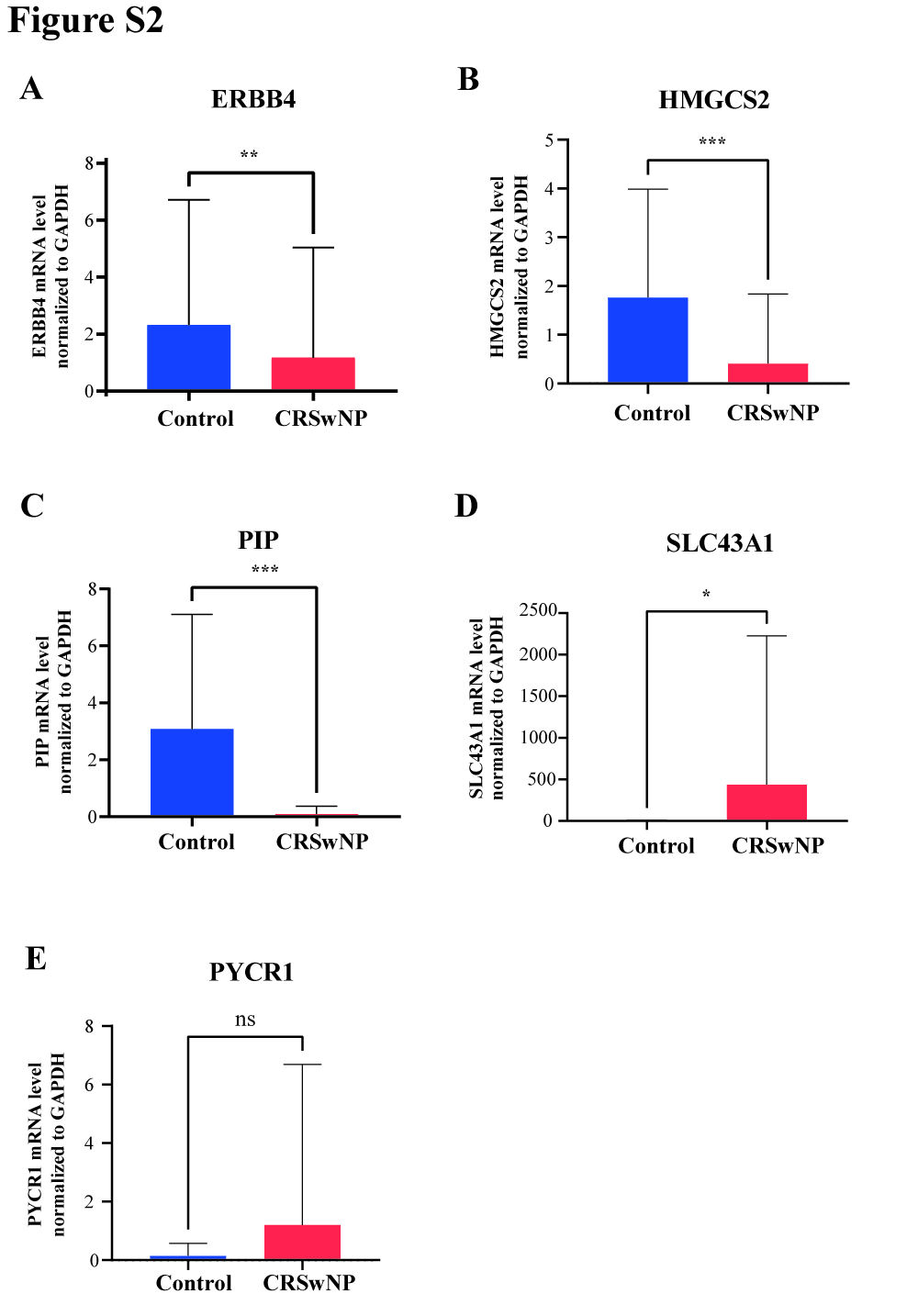

Supplement: Supplementary file 3 [file Image2.tif]

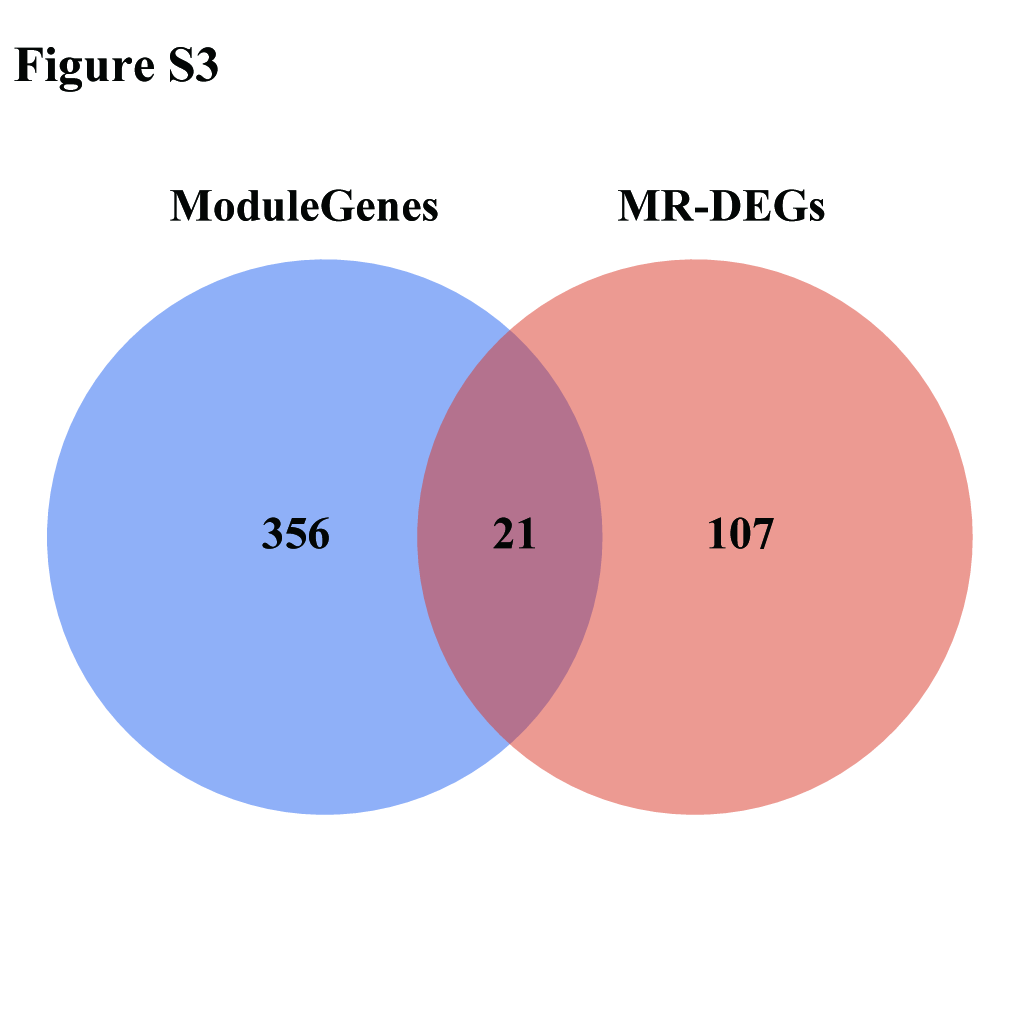

Supplement: Supplementary file 4 [file Image3.tif]
